# Fibroblast Growth Factor 2 lethally sensitizes cancer cells to stress‐targeted therapeutic inhibitors

**DOI:** 10.1002/1878-0261.12402

**Published:** 2018-12-03

**Authors:** Matheus H. Dias, Cecília S. Fonseca, Julianna D. Zeidler, Layra L. Albuquerque, Marcelo S. da Silva, Eduardo Cararo‐Lopes, Marcelo S. Reis, Vincent Noël, Edmilson O. dos Santos, Ian A. Prior, Hugo A. Armelin

**Affiliations:** ^1^ Center of Toxins Immune‐response and Cell Signaling (CeTICS) and Laboratório Especial de Ciclo Celular (LECC) Instituto Butantan São Paulo Brazil; ^2^ Cellular and Molecular Physiology Institute of Translational Medicine University of Liverpool UK; ^3^ Instituto de Química Universidade de São Paulo Brazil

**Keywords:** FGF2, malignant phenotype, MAPK‐ERK1/2, mitogenic signaling, stress‐targeted therapy, synthetic lethality

## Abstract

In malignant transformation, cellular stress‐response pathways are dynamically mobilized to counterbalance oncogenic activity, keeping cancer cells viable. Therapeutic disruption of this vulnerable homeostasis might change the outcome of many human cancers, particularly those for which no effective therapy is available. Here, we report the use of fibroblast growth factor 2 (FGF2) to demonstrate that further mitogenic activation disrupts cellular homeostasis and strongly sensitizes cancer cells to stress‐targeted therapeutic inhibitors. We show that FGF2 enhanced replication and proteotoxic stresses in a K‐Ras‐driven murine cancer cell model, and combinations of FGF2 and proteasome or DNA damage response‐checkpoint inhibitors triggered cell death. CRISPR/Cas9‐mediated K‐Ras depletion suppressed the malignant phenotype and prevented these synergic toxicities in these murine cells. Moreover, in a panel of human Ewing's sarcoma family tumor cells, sublethal concentrations of bortezomib (proteasome inhibitor) or VE‐821 (ATR inhibitor) induced cell death when combined with FGF2. Sustained MAPK‐ERK1/2 overactivation induced by FGF2 appears to underlie these synthetic lethalities, as late pharmacological inhibition of this pathway restored cell homeostasis and prevented these described synergies. Our results highlight how mitotic signaling pathways which are frequently overridden in malignant transformation might be exploited to disrupt the robustness of cancer cells, ultimately sensitizing them to stress‐targeted therapies. This approach provides a new therapeutic rationale for human cancers, with important implications for tumors still lacking effective treatment, and for those that frequently relapse after treatment with available therapies.

**Abbreviations**
DDR: DNA damage response
ESFT: Ewing's sarcoma family tumors
FGF2: fibroblast growth factor 2
MAPK: mitogen‐activated protein kinase
PI: propidium iodide
UPR: unfolded protein response

## 1 Introduction

Several cellular stress‐response pathways are frequently mobilized in malignant cells to cope with an aggressive and highly proliferative phenotype. Identification and targeting of cancer cells‐specific vulnerabilities resulting from these stresses is a promising therapeutic approach, particularly for cancers in which the driver oncogene is not clinically druggable. For instance, gain‐of‐function mutations and overexpression of RAS family members (KRAS, HRAS, and NRAS) are among the most prevalent oncogenic lesions in human cancers (Prior *et al*., 2012), and high levels of Ras activity are necessary to maintain the transformed phenotype in some Ras‐driven cancers (Singh *et al*., 2009). Similar oncogene addiction is also described for Ewing's sarcoma family tumors (ESFT), which are a group of childhood and adolescence poorly differentiated cancers, arising from bone and soft tissues (Biswas and Bakhshi, 2016). The (11;22) (q24;q12) chromosomal translocation encoding the fused transcription factor EWS‐FLI‐1 is present in approximately 85% of all Ewing's sarcoma family tumor specimens and is established as the driver oncogenic lesion in these tumors (Toomey *et al*., 2010). In common with Ras‐driven tumors, Ewing's sarcoma family tumors display a poor prognosis at metastatic stage (cure rate of 20–40%) and the lack of clinically effective targeted therapies (Gaspar *et al*., 2015). Hence, selective targeting of stress‐response pathways supporting Ras and EWS‐FLI‐1‐driven tumorigenesis might be game‐changing for the therapy of these aggressive malignancies.

Enhanced DNA damage and replication stress are probably the best characterized and exploited stresses resulting from malignant transformation induced by Ras, EWS‐FLI‐1, and other oncogenes (Hills and Diffley, 2014). Genotoxic agents such as ionizing radiation, cisplatin, and gemcitabine are widely used in cancer therapy aiming to push tumor DNA damage/replication stress over a lethal threshold (Swift and Golsteyn, 2014). More recently, checkpoint inhibition was shown to increase the cell death induced by these genotoxic agents (Prevo *et al*., 2012).

The enhanced proteotoxic stress frequently found in malignant cells is also a clinical target in cancer therapy. Proteasome inhibition resulting in lethal proteotoxic stress is a protagonist treatment for some hematological cancers (Csizmar *et al*., 2016). Combined induction of protein misfolding further enhanced proteotoxic stress, increasing the cytotoxicity of proteasome inhibition *in vitro* and *in vivo* (Neznanov *et al*., 2011). Drawbacks of these stress‐targeted therapies include the high overall toxicity and acquired resistance of genotoxic agents and proteasome inhibitors like bortezomib, limiting the therapeutic window and efficacy of these approaches (Cavaletti and Jakubowiak, 2010; Kalal *et al*., 2017). Altogether, these observations point that overload of replication or proteotoxic stress, especially in combination with the respective sensitizing inhibition, might efficiently target cancer cells‐specific vulnerabilities. Therefore, identification of effective combinations ‘Stress induction/sensitizing inhibition’ targeting selectively malignant cells is paramount.

In this regard, exogenous administration of the fibroblast growth factor 2 (FGF2) might be a viable alternative to overload cellular stress pathways in cancer cells. FGF2 is the seminal member of a large family of signaling factors of undisputed importance for neurogenesis, morphogenesis, wound healing, and angiogenesis, among other functions (Armelin, 1973; Itoh and Ornitz, 2011). Despite the many different protumor roles attributed to FGF2 signaling (reviewed by Turner and Grose, 2010), a set of articles unequivocally demonstrate that FGF2 can also induce cytostatic and cytotoxic responses in different cancer cells, both *in vivo* and *in vitro* (Fogarty *et al*., 2007; Sturla *et al*., 2000; Wang *et al*., 1998; Williamson *et al*., 2004). In this last context, we have also previously shown that FGF2 restrains the proliferation of murine malignant cells, in which wild‐type Kras is highly amplified and overexpressed (Costa *et al*., 2008; Salotti *et al*., 2013). Because FGF2 is an activator of mitogenic signaling pathways, we hypothesized that the toxicity induced by this growth factor in cancer cells might also intensify the mobilization of stress pathways, further increasing their dependency on these pathways for cell viability.

Here, we tested whether FGF2 can selectively sensitize cancer cells to stress‐targeted therapeutic inhibitors. We found that in K‐Ras‐driven mouse Y1 malignant cells FGF2 stimulation disrupts proteostasis and enhances tonic replication stress and DNA damage response (DDR) activation. Concomitant proteasome or checkpoint inhibition induced cell death in a K‐Ras‐dependent manner. Importantly, in human ESFT cells, combined FGF2 signaling activation and sublethal doses of proteasome or checkpoint inhibitors also triggered cell death. Moreover, FGF2 induced sustained MAPK‐ERK1/2 overactivation in Y1 and ESFT cells, and K‐Ras depletion or late pharmacological mitogen‐activated protein kinase (MAPK) inhibition, respectively, prevented FGF2 sensitization to these stress‐targeted inhibitors. These findings indicate that further activation of mitogenic signaling can be employed to overload stress‐response pathways selectively in cancer cells, disrupting their homeostatic robustness, and increasing the cytotoxicity of stress‐targeted therapies.

## 2 Materials and methods

### 2.1 Cell lines, cell culture, and treatments

The Y1 murine adrenocortical carcinoma cell line was obtained from ATCC. Y1 cells were grown at 37 °C in a 5% CO_2_ atmosphere in Dulbecco's modified Eagle's medium (DMEM; Gibco, Waltham, MA, USA) supplemented with 10% FBS. The Y1D1 subline (Schwindt *et al*., 2003) was cultured in the same conditions as Y1, and the growth medium was supplemented with 0.1 mg·mL^−1^ geneticin (G418; Invitrogen, Carlsbad, CA, USA). Whenever G0/G1 synchronization by serum starvation was necessary DMEM/FBS medium was removed, plates were washed with PBS, and cells were grown in FBS‐free DMEM for 48 h prior any stimulation. ESFT cells (A673, RD‐ES, SK‐N‐MC, and TC‐32) were kindly given by Professor Susan Burchill. A673 and SK‐N‐MC cells were grown in DMEM 10% FBS, TC‐32 cells were grown in RPMI 1640 10% FBS, and RD‐ES cells were grown in RPMI 1640 15% FBS. Where indicated, cells were treated with recombinant human FGF2 protein (Abcam ab9596, Cambridge, MA, USA); colchicine (Sigma C9754, St. Louis, MO, USA); the MEK inhibitor U0126 (Promega V1121, Madison, WI, USA); bortezomib (#S1013); ATM inhibitor KU55933 (#S1092); ATR inhibitor VE‐821 (#S8007); p38 inhibitor SB202190 (S1077); and MEK inhibitors selumetinib (#S1008) and trametinib (S2673); all these last inhibitors from Selleckchem (Houston, TX, USA).

### 2.2 Cell cycle analysis

For bromodeoxyuridine (BrdU)/propidium iodide cell cycle analyses, after the indicated treatments, cells were resuspended and fixed in ice‐cold 75% ethanol in PBS overnight at 4 °C. BrdU was added at 50 μm for 30 min before harvesting. Fixed cells were treated with 2 m HCl and 0.5% Tween‐20 for 15 min for DNA denaturation and then washed sequentially with 0.1 m sodium tetraborate (pH 9.5) and ice‐cold PBS. Cells were incubated with Alexa Fluor^®^ 488 anti‐BrdU (Invitrogen B35130) and subsequently treated with 10 mg·mL^−1^ RNase A and stained with 50 mg·mL^−1^ propidium iodide in PBS for 20 min before analysis in the flow cytometer.

For all flow cytometer experiments, data were acquired with Attune NxT flow cytometer (Life Technologies, Carlsbad, CA, USA) and analyzed with flowjo V.10 software (Treestar, Inc., Ashland, OR, USA). At least 20 000 cells per sample were analyzed.

### 2.3 Histones on cell cycle assays

For phospho‐histone H3 (S10) or phospho‐H2AX‐S139 (γ‐H2AX) stains along cell cycle, after treatment cells were fixed as described above, washed in PBS, and incubated for 1 h with the conjugated histone antibodies (histone H3 S10 Millipore 06‐570‐AF488, St Louis, MO, USA or γ‐H2AX Thermo Fisher 53‐9865‐82, Waltham, MA, USA) Samples were then treated with 10 mg·mL^−1^ RNase A and stained with 50 mg·mL^−1^ propidium iodide in PBS for 20 min prior to analysis by flow cytometry.

### 2.4 Protein per cell assay

To measure protein/cell, 2.5 × 10^4^ cells per 35‐mm dish were plated, let to adhere overnight and then serum starved as described for synchronization. After 48 h, cells were stimulated with FBS in presence or absence of FGF2 as indicated for each experiment. For each condition and time point, we harvested three plates for counting cells, as described below for growth curves, and three plates for measuring total protein concentration. To estimate the amount of protein per cell, we measured the amount of protein from each plate using Bradford method and divided by the number of cells counted from each plate of the same time point and condition.

### 2.5 Western blots

Antibodies for western blot were as follows: IRE1α (3294; Cell signaling, Danvers, MA, USA), Bip (3183; Cell signaling), phospho‐S6 Ser235/236 (4856; Cell Signaling), phospho‐eIF4E Ser209 (9741; Cell Signaling), α‐tubulin (sc‐8035; Santa Cruz, Santa Cruz, CA, USA), phospho‐H2AX S139 (ab11174; Abcam), ChK1 (ab47574; Abcam), phospho‐ChK1 S345 (sc‐17922; Santa Cruz), phospho‐ChK2 T383 (ab59408; Abcam), p38 (9212; Cell Signaling); phospho‐p38 T180/Y182 (sc‐15852‐R; Santa Cruz), p21 (sc‐397; Santa Cruz), HPRT (sc‐20975; Santa Cruz), K‐Ras (sc‐30; Santa Cruz), actin (ab6276; Abcam), phospho‐ERK Thr202/204 (4370 and 9101; Cell Signaling), and ERK (4695 and 9102; Cell Signaling). Analysis was performed by standard methods using enhanced chemiluminescence or fluorescence. Images were obtained using Uvitec Alliance 9.7 documentation system (Uvitec, Cambridge, UK) or Odyssey system (Licor, Cambridge, UK) according to the manufacturer's settings.

### 2.6 Cell death assay

For AnnexinV/propidium iodide (PI) staining, cells were plated on 35‐mm dishes and treated as described for each experiment. Then, culture media were collected and reserved, plates were washed with 450 μL of PBS, which was also reserved on the respective tubes, and cells were released with 150 μL of trypsin for up to 5 min. Cells were then suspended and homogenized with the respective culture medium/PBS. The volume of all the suspensions were adjusted to 2 mL. Two hundred and fifty microlitre of each cell suspension was mixed 1 : 1 with 2× Annexin V binding buffer (300 mm NaCl, 2 mm MgCl_2_, 10 mm KCl, 3.6 mm CaCl_2_, 20 mm HEPES pH 7.4) containing 1 : 10 000 AnnexinV‐FITC (produced and kindly given by Shankar Varadarajan's laboratory). After 10 min, 5 μL of PI 50 μg·mL^−1^ was added to each tube and mixed by inversion. Fixed volumes of these cell suspensions were then analyzed using Attune NxT Flow cytometer (Thermo Fisher Scientific's Invitrogen) allowing combined determination of cell viability and total amount of cells per plate whenever necessary. At least 20 000 cells were analyzed for each individual sample.

### 2.7 Detection of BrdU foci under native DNA conditions

For detection of long fragments of single‐stranded DNA (ssDNA), characteristics of replication stress, exponentially growing cells in coverslips incorporated BrdU at 50 mm for 48 h into DNA. After that, we washed the coverslips and added fresh DMEM with or without 10 ng·mL^−1^ FGF2 for 24 h. To ensure that all cells incorporated BrdU, one additional coverslip for each condition analyzed was prepared to be subjected to DNA denaturation using 2 m HCl. Next, cells were fixed using 4% of paraformaldehyde in PBS and permeabilized with 0.2% Triton‐X 100. BrdU was detected (when accessible) using α‐BrdU‐rat (ab6326 Abcam) followed by secondary antibody goat anti‐rat conjugated to Alexa Fluor 488 (A‐11006 Thermo Scientific). Stained coverslips were mounted with VECTASHIELD^®^ Mounting Medium with DAPI (Vector Labs). Images were captured using Olympus BX51 fluorescence microscope coupled with a digital camera (XM10; Olympus) and analyzed using olympus cell f software (version 5.1.2640, Tokyo, Japan). At least 65 cells were analyzed per coverslip.

### 2.8 Cas9‐mediated K‐Ras depletion

To deplete K‐Ras expression in Y1 cells, we designed and tested five different specific gRNAs against the k‐ras gene using CRISPR design tool (http://crispr.mit.edu/). A scramble sequence was also designed for control. Sequences are shown below (Table 1).

**Table 1:** gRNA sequences

1. K‐Ras	5′ CTCCCGCGCCATTTCGGACC 3′
2. K‐Ras	5′ CCTGAGGCGCGGCGGCTCCG 3′
3. K‐Ras	5′ AGATATTCACCATTATAGGT 3′
4. K‐Ras	5′ AAGAGGAGTACAGTGCAATG 3′
5. K‐Ras	5′ CTGAATTAGCTGTATCGTCA 3′
Scramble	5′ GCACTACCAGAGCTAACTCA 3′

Oligos were cloned into LentiGuide‐Puro plasmid (a gift from Feng Zhang, Addgene plasmid # 52963) according to described by Sanjana *et al*. (2014). For lentivirus production, LentiGuide‐Puro constructs, psPAX2 (a gift from Didier Trono, Addgene plasmid # 12260) and pCMV‐VSV‐G (a gift from Robert Weinberg, Addgene plasmid # 8454), were transfected into HEK293T cells using lipofectamine 3000 reagent according to manufacturer's protocol. Forty‐eight hours after transfection, viral supernatants were collected and filtered. Y1 cells were then cotransduced with the LentiCas9‐Blast and the individual LentiGuide‐Puro constructs in presence of 8 μg·mL^−1^ of polybrene (sc‐134220; Santa Cruz Biotechnology). Forty‐eight hours after Y1 transduction, cells were selected with 3 μg·mL^−1^ of puromycin and 7 μg·mL^−1^ of blasticidin for 7 days before testing knock‐out efficiency. For all experiments here, we used the subline 4 (hereafter Y1ΔK), which displayed the lower levels of K‐Ras expression.

### 2.9 Clonogenic and viability assays

The indicated amounts of cells per well were plated on six wells or p60 plates (Fig. 4B only), let to adhere overnight and then treated as described. After that, the culture media were replaced every other day until the endpoint. Cells were then washed with PBS, fixed, and stained in a fixing/staining solution (0.05% crystal violet, 1% formaldehyde, 1% methanol in PBS) and washed abundantly. Images were acquired using GelCount colony analyzer (Oxford Optronix, Oxford, UK).

### 2.10 Growth curves

At day 0, 3 × 10^4^ cells per 35‐mm culture dish were plated in DMEM/FBS medium with or without FGF2. At the indicated days, cells were harvested in triplicates, fixed in formaldehyde 3.7%, diluted in phosphate‐buffered solution (PBS), and stored. The medium of reminiscent plates was changed in every 2 days. Cells were later counted in a Z2 Beckman Coulter^®^ counter.

### 2.11 Nonadherent proliferation assay

At day 0, 1 × 10^4^ cells per well were plated on ultra‐low attachment 96‐well plates (Corning CLS3474, Corning, NY, USA). Relative cell viability/proliferation was measured after 1 day, to set up a baseline, and after 10 days using CellTiter 96 AQ_ueous_ (Promega G3582) according to the manufacturer's protocol. A least nine wells per cell were assayed at each time point.

### 2.12 Statistical analyses

Bar graphs with two columns were analyzed with paired Student's *t*‐test, and bar graphs with three or more columns were analyzed by one‐way ANOVA followed by multiple comparison post‐test. Growth curves were analyzed by two‐way ANOVA followed by multiple comparison post‐test. All graphics and statistical analyses were performed using graphpad prism 7 software (GraphPad Software, La Jolla, CA, USA).

## 3 Results

### 3.1 FGF2 impairs cell cycle progression in K‐Ras‐driven cancer cells

We previously showed that FGF2 triggers G0/G1→S transition but irreversibly restrains the proliferation of K‐Ras‐driven Y1 malignant cells (Costa *et al*., 2008). Y1 cells are poorly synchronized by serum starvation. Hence, to address FGF2 effects along cell cycle progression accurately, we initially used the Y1 D1 subline. These cells, which we described elsewhere (Schwindt *et al*., 2003), display strict control of quiescence/proliferation switch in response to serum and are phenotypically identical to parental Y1 cells regarding karyotype, K‐Ras overexpression, and malignant phenotype.

**Figure 1:**
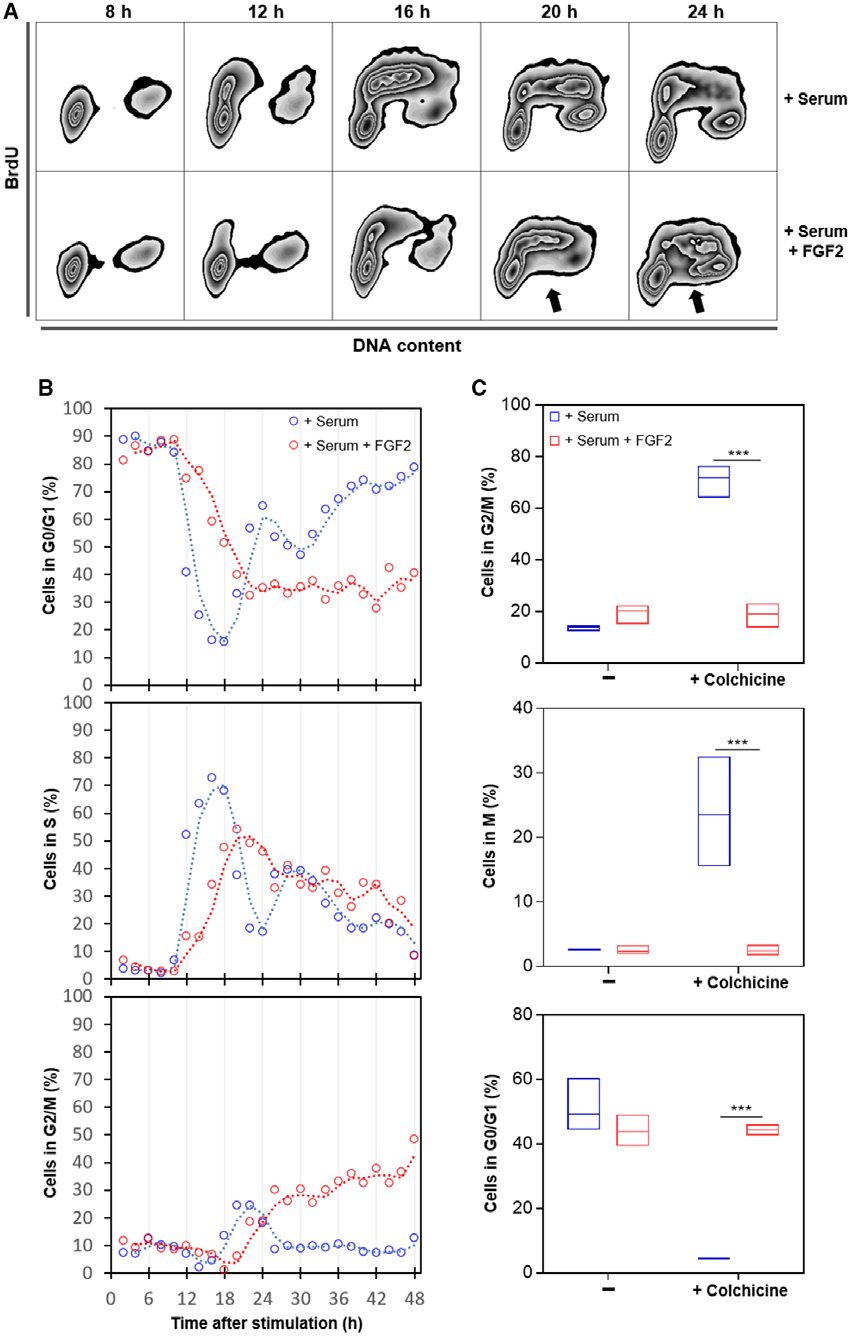
FGF2 impairs cell cycle progression in K‐Ras‐driven cancer cells. Serum‐starved Y1D1 cells were stimulated by 10% serum with or without 10 ng·mL^−1^ FGF2 to re‐entry the cell cycle. Cells were subjected to a BrdU pulse 30 min before sample collection (every two hours). (A) Representative zebra plot flow cytometry data of BrdU‐stained cells versus DNA content at the indicated times after stimulation comparing cell cycle re‐entry and progression with or without FGF2. BrdU was added at 50 μm for 30 min before harvesting. The arrows indicate BrdU‐unlabeled S‐phase cells. (B) Time‐course flow cytometry analyses comparing the progression along cell cycle phases from 2 to 48 h after stimulation by serum with or without FGF2. BrdU was added at 50 μm for 30 min before harvesting. (C) Quantifications of flow cytometry data showing phospho‐histone H3 (S10) and DNA content double stain. The proportions of cells in each phase were measured 24, 28, 32, and 36 h after stimulation with serum or serum + FGF2 in presence or absence of 2 μm colchicine. Mitotic cells were addressed by chromatin condensation indicated by phospho‐histone H3 (S10)‐positive stain. Asterisks indicate significant differences. ***P ≤ 0.001 (one‐way ANOVA of variance followed by multiple comparison post‐test) (*n* = 4).

Initially, we followed cell cycle progression of G0/G1‐arrested Y1D1 cells after serum stimulation +/− FGF2, collecting samples every 2 h, with a 30‐min pulse of BrdU uptake into DNA, immediately before cell harvesting (Fig. 1A,B). Flow cytometry results showed that FGF2 delayed both cell entry in and progression through S phase (Fig. 1B, upper and middle panels). In FGF2‐treated samples, after 20 h of stimulation, we observed BrdU‐unlabeled S‐phase cells, indicating DNA synthesis arrest (Fig. 1A arrows). Moreover, between 24 and 48 h, we found a parallel decrease in S‐phase and accumulation in G2/M subpopulation (Fig. 1B, middle and lower panels). This accumulation likely was due to G2 arrest, since mitosis blockage by colchicine induced G2‐ and M‐phase accumulation in serum‐stimulated but not in FGF‐2‐treated cells between 24 and 36 h (Fig. 1C, top and middle panels). Notably, about 40% of the FGF2‐stimulated cells remained in G0/G1 phase irrespective of colchicine addition (Fig. 1B,C, bottom panel), indicating that many of the FGF2‐stimulated cells were not even able to leave G1 phase. These results showed that FGF2 compromises cell fitness, impairing the progression throughout the cell cycle in these Ras‐driven malignant cells.

### 3.2 FGF2 exacerbates replication stress and sensitizes K‐Ras‐driven cancer cells to checkpoint inhibition toxicity

**Figure 2:**
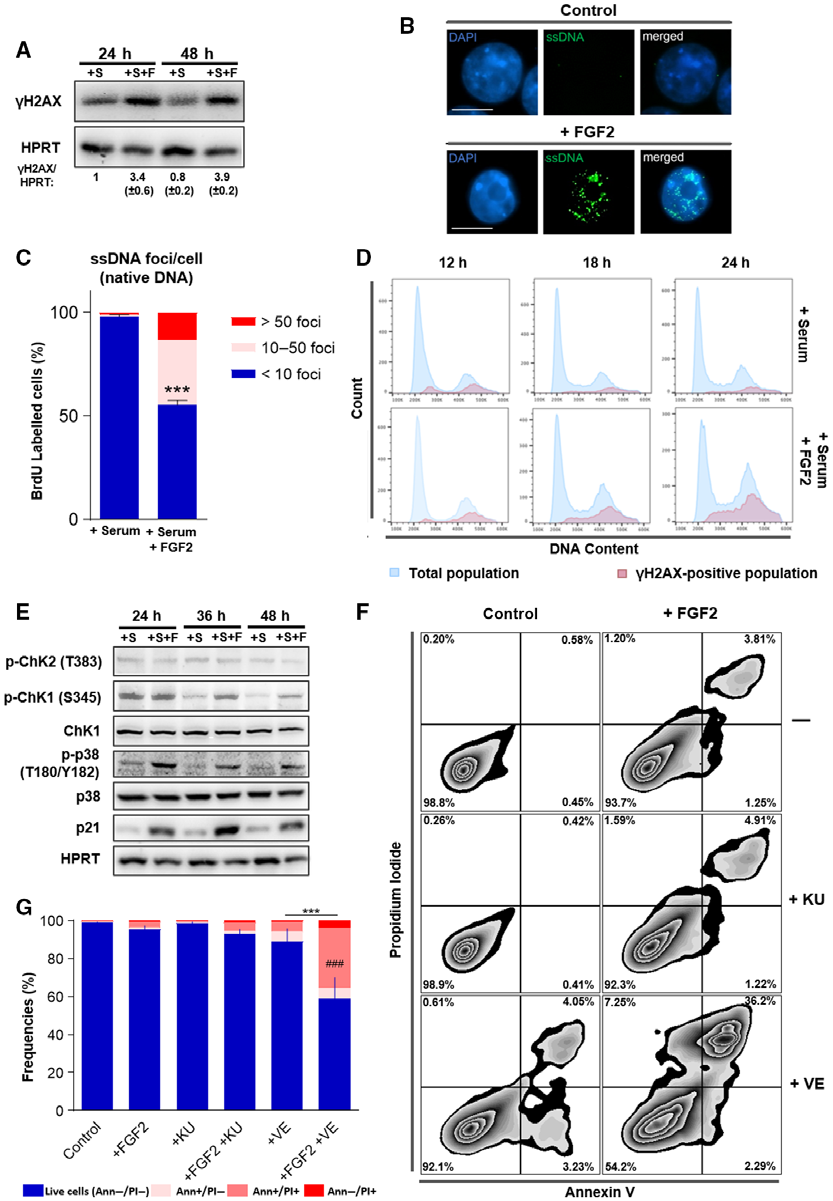
FGF2 reinforces replication stress in K‐Ras‐driven cancer cells increasing ATR‐checkpoint inhibition toxicity. (A) Western blots comparing the levels of phospho‐H2AX histone (γ‐H2AX) in Y1 cells. Cells were serum starved and then restimulated with 10% serum (+S) or 10% serum + 10 ng·mL^−1^ FGF2 (+S+F) for the indicated times. HPRT was used as a loading control. Quantifications were performed using uvitec alliance 9.7 software (Uvitec). (B) Representative immunofluorescence detection of single‐stranded DNA (ssDNA) foci under native conditions. Fifty millimolar of BrdU was incorporated to Y1 cells for 48 h and then washed out. Cells were grown for additional 24 h in complete media with or without 10 ng·mL^−1^ FGF2 and then stained for BrdU (green) and DNA (blue) under nondenaturing conditions. White bars correspond to 10 μm. (C) Quantification of ssDNA foci per cell from the experiments described in (B). Error bars indicate mean ± SD. This assay was performed in triplicate with measurement of at least 65 cells per replicate (*n* = 65/assay). Asterisks indicate statistically significant differences. ***P ≤ 0.001 (paired Student's *t*‐test). (D) Representative histogram flow cytometry data comparing serum and serum + FGF2 stimulation regarding γ‐H2AX distribution along the cell cycle phases. Y1 cells were serum starved and then restimulated by 10% serum with or without 10 ng·mL^−1^ FGF2 for the indicated times. (E) Western blots comparing the levels of the DDR and checkpoint markers phosphorylated Chk1 (p‐Chk1), phosphorylated Chk2 (p‐Chk2), phosphorylated p38 MAPK (p‐p38), and p21 in Y1 cells restimulated by 10% serum (+S) or 10% serum + 10 ng·mL^−1^ FGF2 (+S+F) for the indicated times after serum starvation. Total Chk1, p38, and HPRT were used as loading controls. (F) Representative zebra plot flow cytometry data of Y1 cells growing in complete media in presence or absence of 10 ng·mL^−1^ FGF2 for 48 h, with concomitant addition of 5 μm of the ATM inhibitor KU‐55933 (KU) or the ATR inhibitor VE‐821 (VE). Annexin V/PI double stain was used to address cell death. (G) Quantification of the experiments described in (F). Error bars indicate mean ± SD of live cells (*n* = 3, from independent experiments). Asterisks indicate statistically significant differences. ***P ≤ 0.001 (one‐way ANOVA of variance followed by multiple comparison post‐test). ^#^Significant differences from the FGF2‐treated sample. ^###^P ≤ 0.001

The S‐phase cells displaying DNA synthesis arrest in the flow cytometry data (Fig. 1A arrows) suggested that FGF2 induced replication stress in this K‐Ras‐driven cell model. As unresolved replication arrest generates double‐strand breaks, we measured the levels of the DNA damage marker phospho‐H2AX histone (γ‐H2AX) (Gagou *et al*., 2010) in Y1 cells stimulated by serum +/− FGF2. Serum‐stimulated cells exhibited moderate levels of γ‐H2AX, and these levels increased over 3.5‐fold by FGF2 stimulation (Fig. 2A). As a more specific readout of replication stress, we incorporated the thymidine analogue BrdU to these cells and measured ssDNA foci at native conditions. FGF2 stimulation resulted in about 45% of the cells showing more than 10 ssDNA foci after 24 h, comparing to less than 3% of the control cells (Fig. 2B,C). To further confirm that such DNA damage results from replication stress, we analyzed the distribution of γ‐H2AX‐positive cells along the cell cycle at three different time points after stimulation. Corroborating the above results, serum‐stimulated cells displayed moderate γ‐H2AX staining, and FGF2 increased γ‐H2AX‐positive cell population 18 and 24 h after stimulation (Fig. 2D). In all samples, γ‐H2AX‐positive cells were almost exclusively found in S and G2 phases, pointing that the observed DNA damage in these cells is likely a consequence of replication stress.

These observations prompted us to probe for the engagement of the DDR. We reasoned that DDR and checkpoint activation might contribute to the observed cell cycle arrest triggered by FGF2 in these cells, as a protective response against FGF‐induced replication stress. We first assessed the activation status of classical DDR and checkpoint effector proteins, that is, Chk 1 and 2, p21, and p38 (reviewed by Harper and Elledge, 2007). The results showed that FGF2 does not alter the levels of active Chk2; however, FGF2 increased the levels of phosphorylated Chk1, p38, and p21 comparing to serum‐stimulated samples (Fig. 2E). These results confirmed that FGF2‐induced replication stress triggers DDR and checkpoint activation in these cells.

Checkpoint inhibition prevents cell cycle arrest induced by DNA damaging chemotherapy, forcing cancer cells into a defective mitosis and consequent cell death (Huntoon *et al*., 2013). To assess whether combined FGF2 signaling and checkpoint inhibition could lead to this same outcome, we focused on ATM and ATR, the two major kinases controlling checkpoint response (Harper and Elledge, 2007). We treated cells with specific ATR (VE‐821) (Reaper *et al*., 2011) and ATM (KU‐55933) (Hickson *et al*., 2004) pharmacological inhibitors for 48 h in presence or absence of FGF2 and measured cell death on the flow cytometer. FGF2 only modestly increased cell death compared to serum‐stimulated control samples. ATM inhibition had no significant effect on cell death with or without FGF2. ATR inhibition alone moderately increased cell death. Strikingly, the association of VE‐821 and FGF2 induced over 40% of cell death after 48 h (Fig. 2F,G).

Altogether, these results indicate that Y1 cells, as proposed for other cancer models, deal with chronic replication stress, and rely on checkpoint activation for cell survival. FGF2 signaling upregulated such basal replication stress leading to cell cycle arrest and, at the same time, increasing checkpoint dependence. Thus, FGF2 sensitizes this K‐Ras‐driven malignant model to cell death induced by ATR‐mediated checkpoint inhibition.

### 3.3 FGF2 induces proteotoxic stress and sensitizes K‐Ras‐driven cancer cells to bortezomib cytotoxicity

**Figure 3:**
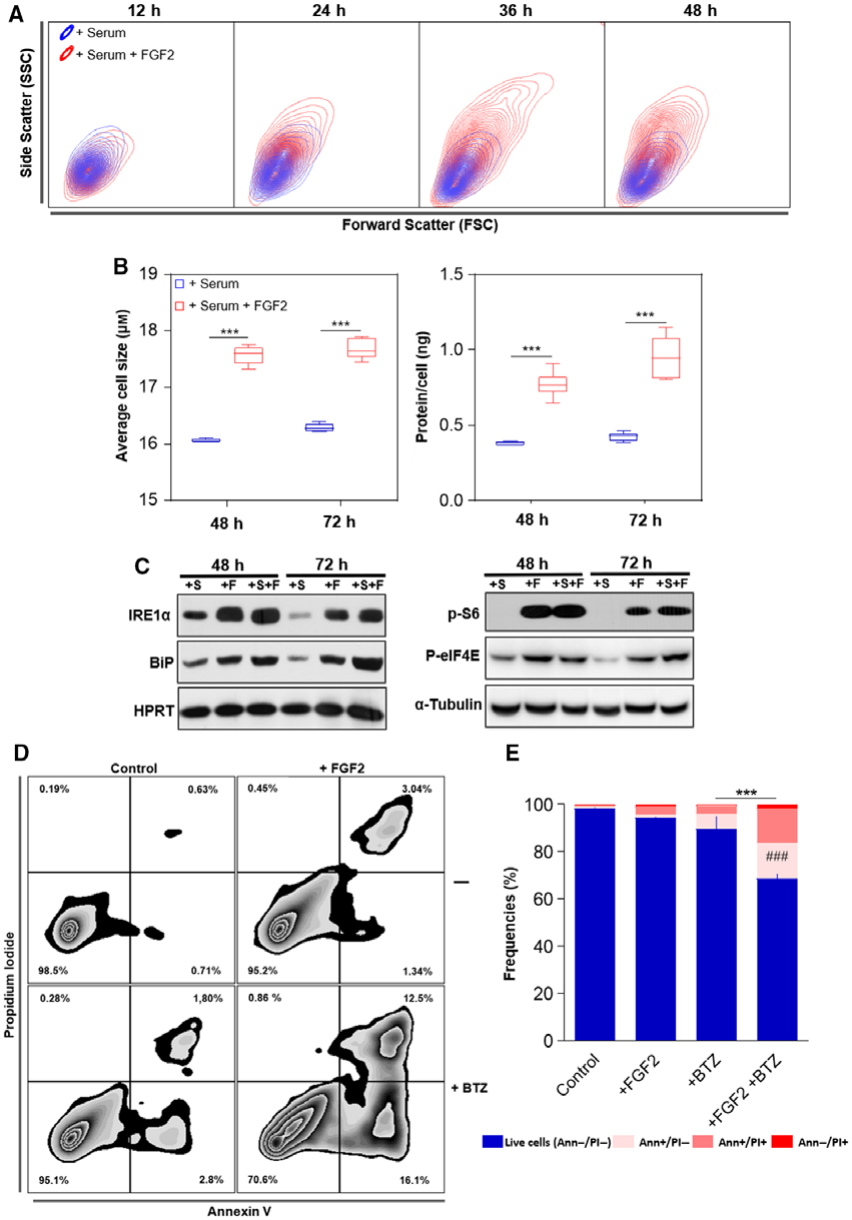
FGF2 disrupts the proteostasis and sensitizes K‐Ras‐driven cancer cells to bortezomib toxicity. (A) Representative contour plot flow cytometry data from samples of Fig. 1B comparing serum and serum + FGF2 stimulation regarding cell size (forward scatter) and internal complexity (side scatter) along the time. (B) Measurements of the average cell size and the amount of protein per cell comparing serum and serum + FGF2 stimulated cells. Y1 cells were serum starved and then restimulated by the indicated times. Asterisks indicate significant differences. ***P ≤ 0.001 (one‐way ANOVA of variance followed by multiple comparison post‐test) (*n* = 6, from independent experiments). (C) Western blots comparing the levels of the UPR markers IRE1α and Bip (left panel); and the phosphorylated forms of S6 ribosomal protein (p‐S6) and eukaryotic translational initiation factor 4E (p‐EIF4E; right panel) among the different stimuli. Y1 cells were serum starved and then restimulated with 10% serum (+S); 10 ng·mL^−1^ FGF2 (+F); or both (+S+F) for the indicated times. HPRT and α‐tubulin were the used as loading controls. (D) Representative zebra plot flow cytometry data of Y1 cells growing in complete media in presence or absence of 10 ng·mL^−1^ FGF2 for 96 h, with the addition of 20 nm bortezomib (BTZ) in the last 72 h. Annexin V/PI double stain was used to address cell death. (E) Quantification of the experiments described in (D). Error bars indicate mean ± SD of live cells (*n* = 3, from independent experiments). Asterisks indicate statistically significant differences. ***P ≤ 0.001 (one‐way ANOVA of variance followed by multiple comparison post‐test). ^#^Significant differences from the FGF2‐treated sample. ^###^P ≤ 0.001

In agreement with our previous report (Costa *et al*., 2008), flow cytometry data from FGF2‐treated cells presented on Fig. 1 showed increased cell size (FSC) and internal complexity (SSC) comparing to serum‐stimulated control cells (Fig. 3A). To further investigate this dual effect of FGF2 blocking proliferation but keeping cells growing, we stimulated serum‐starved Y1 cells with serum +/− FGF2 and measured average size and the amount of protein per cell. FGF2‐stimulated cells displayed increased cell size and about twice the amount of protein/cell measured on serum‐stimulated samples after 48 and 72 h (Fig. 3B). These results indicated that although FGF2 triggered cell cycle arrest in Y1 cells, it stimulated cell growth concerning volume and mass.

The rates of protein synthesis and degradation show physiological fluctuations; however, an optimal balance between these processes is required to warrant cell viability (Walter and Ron, 2011). We then investigated whether this protein overload induced by FGF2 results in proteotoxic stress and, consequently, unfolded protein response (UPR) activation. To this end, we measured the levels of the endoplasmic reticulum kinase IRE1α and the molecular chaperone Bip, two core sensors of UPR, which are upregulated in cells facing proteotoxic stress (Ron and Walter, 2007). We found increased levels of both proteins at 48 and 72 h‐FGF2‐stimulated cells, even in serum‐free media, comparing to serum‐stimulated control samples (Fig. 3C, left). Interestingly, despite this active UPR, FGF2‐stimulated cells displayed high levels of both phosphorylated S6 ribosomal protein and eukaryotic translational initiation factor 4E (eIF4E) (Fig. 3C, right). The phosphorylated forms of these proteins indicate active protein synthesis (Sonenberg and Hinnebusch, 2009), implying that FGF2 aggravates the proteotoxic stress by maintaining active protein synthesis irrespective of an ongoing activated UPR.

The enhanced proteotoxic stress of malignant cells is a clinical target in cancer therapy (Csizmar *et al*., 2016). Hence, we tested whether, beyond the cytostatic effect as a single agent, FGF2 could sensitize Y1 cells to the cytotoxicity of proteasome inhibition. We treated the cells with FGF2 for 24 h and then added bortezomib (BTZ) for additional 72 h before harvesting. We then measured cell death by Annexin V/propidium iodide double stain on the flow cytometer. In absence of FGF2, Y1 cells were very tolerant to 20 nm of BTZ for 72 h, showing almost 90% of live cells. In contrast, the combination of FGF2 and BTZ reduced the percentage of live cells to less than 70%, while FGF2 alone had only minor effects on cell death (Fig. 3D,E). These observations not only show that FGF2 stress response disrupts the proteostasis, but also that it can be combined with proteasome inhibition to trigger cell death in these K‐Ras‐driven cancer cells.

### 3.4 K‐Ras depletion prevents FGF2 toxicity and sensitization to checkpoint or proteasome inhibition in K‐Ras‐driven cancer cells

**Figure 4:**
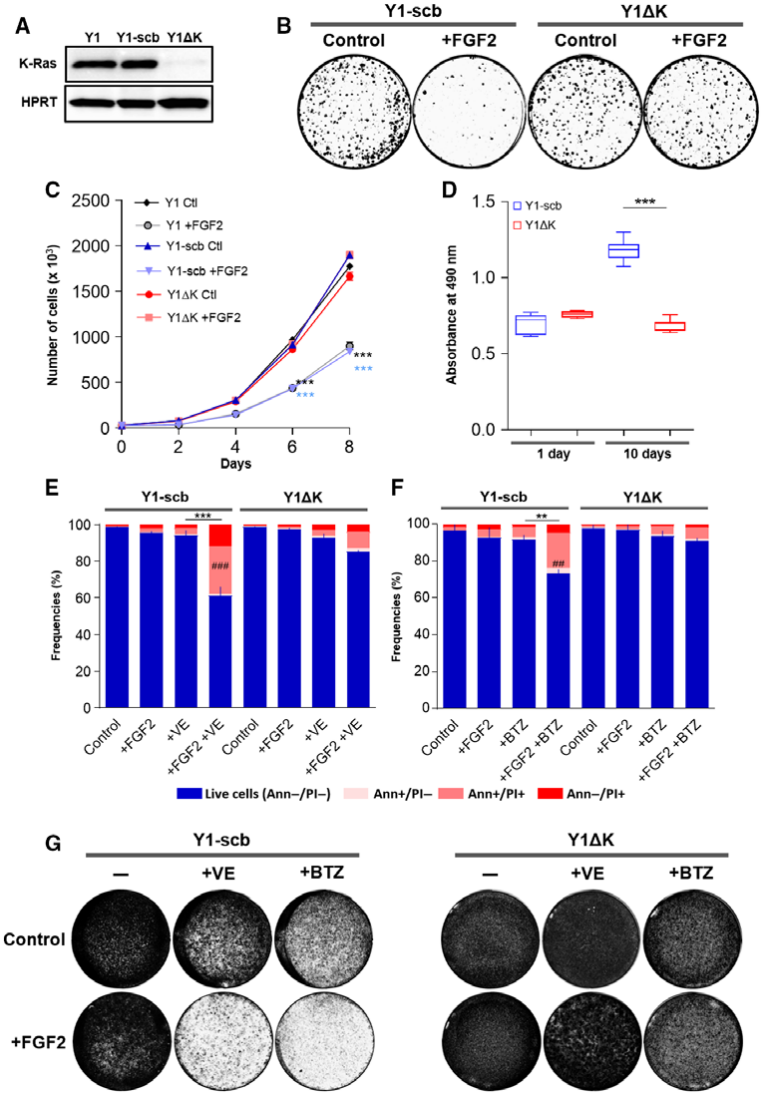
K‐Ras overexpression is required for FGF2 toxicity and sensitization to cell death induced by checkpoint or proteasome inhibition. (A) Western blots comparing the levels of K‐Ras among Y1 parental, Y1‐scb control, and Y1ΔK K‐Ras‐depleted cells. Lysates were prepared from cells growing at complete media. HPRT was used as a loading control. (B) Representative clonogenic assays comparing the viability of Y1‐scb and Y1 ΔK cells. For each cell line, 100 cells·cm^−2^ were plated in complete media in presence or absence of 10 ng·mL^−1^ FGF2, grown for 15 days, and then fixed/stained. Culture media and FGF2 were renewed every 2 or 3 days. (C) Representative growth curves comparing the proliferation of Y1 parental, Y1‐scb control, and Y1 ΔK K‐Ras‐depleted cells. For each cell line, 3 × 10^4^ cells were plated in complete media in presence or absence of 10 ng·mL^−1^ FGF2 and grown for the indicated times. Culture media and FGF2 of the reminiscent plates were renewed at every harvest point. Error bars indicate mean ± SD of technical triplicates. Asterisks indicate significant differences from Y1 control condition. ***P ≤ 0.001 (two‐way ANOVA of variance followed by multiple comparison post‐test). (D) Nonadherent proliferation assay comparing Y1‐scb and Y1 ΔK cells. For each cell line, 1 × 10^4^ cells were plated on ultra‐low attachment 96‐well plates in complete media. Relative cell viability/proliferation was addressed after 1 day, to set up a baseline, and after 10 days to measure proliferation using CellTiter 96 AQ_ueous_ (Promega). At least eight wells per cell were assayed at each time point. Asterisks indicate significant differences. ***P ≤ 0.001 (one‐way ANOVA of variance followed by multiple comparison post‐test). (E) Flow cytometry data of Y1‐scb control, and Y1 ΔK K‐Ras‐depleted cells growing in complete media in presence or absence of 10 ng·mL^−1^ FGF2 for 48 h, with concomitant addition of 5 μm VE‐821 (VE). (F) Flow cytometry data of Y1‐scb control and Y1 ΔK K‐Ras‐depleted cells growing in complete media in presence or absence of 10 ng·mL^−1^ FGF2 for 96 h, with the addition of 20 nm bortezomib (BTZ) in the last 72 h. For (E) and (F), Annexin V/PI double stain was used to address cell death. Error bars indicate mean ± SD of live cells (*n* = 3, from independent experiments). Asterisks indicate statistically significant differences. **P < 0.01 and ***P ≤ 0.001 (one‐way ANOVA of variance followed by multiple comparison post‐test). ^#^Significant differences from the FGF2‐treated sample.^###^P ≤ 0.001 (G) Representative assays comparing the long‐term viability of Y1‐scb and Y1 ΔK cells treated with the combinations of FGF2 and VE‐821 or bortezomib. For each cell line, 2.5 × 10^5^ cells were plated and treated as described in (E) and (F). After the treatments, the stimuli were washed out, the plates were grown in complete media for additional 10 days, and then fixed/stained.

The malignant phenotype of Y1 cells is attributed to the overexpression of the wild‐type K‐Ras protein resulting in high basal levels of K‐Ras‐GTP (Schwab *et al*., 1983). Using the isoform unspecific RasN17 dominant negative, we previously proposed that FGF2 toxicity in Y1 cells depends on high basal levels of K‐Ras‐GTP (Costa *et al*., 2008). To link causally the high levels of K‐Ras protein to FGF2 toxicity and sensitization to stress‐targeted inhibitors, we performed Cas9‐mediated genome editing to deplete K‐Ras protein in these cells. After antibiotic selection, the resultant polyclonal subline (hereafter Y1ΔK) displayed more than 10‐fold reduction in K‐Ras protein levels, comparing to the scramble‐transduced control cells (hereafter Y1‐scb) or the parental Y1 cells (Fig. 4A).

We next enquired whether K‐Ras depletion impacted on viability and proliferation of Y1 cells, as well as its likely protective effect from FGF2 toxicity. Clonogenic assays showed no significant change in cell viability caused by K‐Ras depletion (Fig. 4B). Moreover, K‐Ras depletion prevented FGF2 toxic effects on long‐term viability of Y1 ΔK cells (Fig. 4B). Furthermore, growth curves indicated similar proliferation rates in Y1ΔK cells comparing to both parental Y1 and Y1‐scb control cells, and FGF2 did not impact on cell proliferation of this K‐Ras‐depleted subline (Fig. 4C). Conversely, K‐Ras depletion restrained the proliferation of Y1 ΔK cells under nonadherent growth conditions (Fig. 4D). This set of results shows that K‐Ras depletion elicited robust survival and proliferation in solid substrate but suppressed some malignant phenotype traits in this cell model.

We then investigated whether K‐Ras depletion is sufficient to prevent the cell death induced by simultaneous FGF2 stimulation and proteasome or ATR‐checkpoint inhibition in these cells. To this end, we treated Y1‐scb and Y1 ΔK cells with combinations of FGF2, bortezomib, and VE‐821 in the same regimens described above and measured cell death by flow cytometry. The results for Y1‐scb cells, as expected, were similar to those shown for Y1 parental cells, with the combinations of FGF2 with VE‐821 or bortezomib inducing about 40% and 30% of cell death, respectively (Fig. 4E,F, left). On the other hand, in Y1ΔK cells, K‐Ras depletion largely prevented the cell death induced by the combinations of FGF2 and VE‐821 (Fig. 4E, right) or bortezomib (Fig. 4F, left). To address the effects of these toxicities on long‐term cell viability, we treated both cells using the same regimens described above, washed out FGF2 and the inhibitors, and cultured the cells for additional 10 days. In agreement with the flow cytometry results, combinations of FGF2 and VE‐821 or bortezomib strongly reduced the long‐term viability of Y1‐scb cells (Fig. 4G, left); and K‐Ras depletion fully prevented these toxicities in Y1ΔK cells (Fig. 4G right). Altogether, these data indicated that, in these cells, FGF2 toxicity and the sensitization to proteasome or ATR‐checkpoint inhibition depend on K‐Ras overexpression and malignant phenotype.

### 3.5 FGF2 triggers sustained MAPK‐ERK1/2 overactivation and lethally sensitizes human cancer cells to proteasome and checkpoint inhibitors

**Figure 5:**
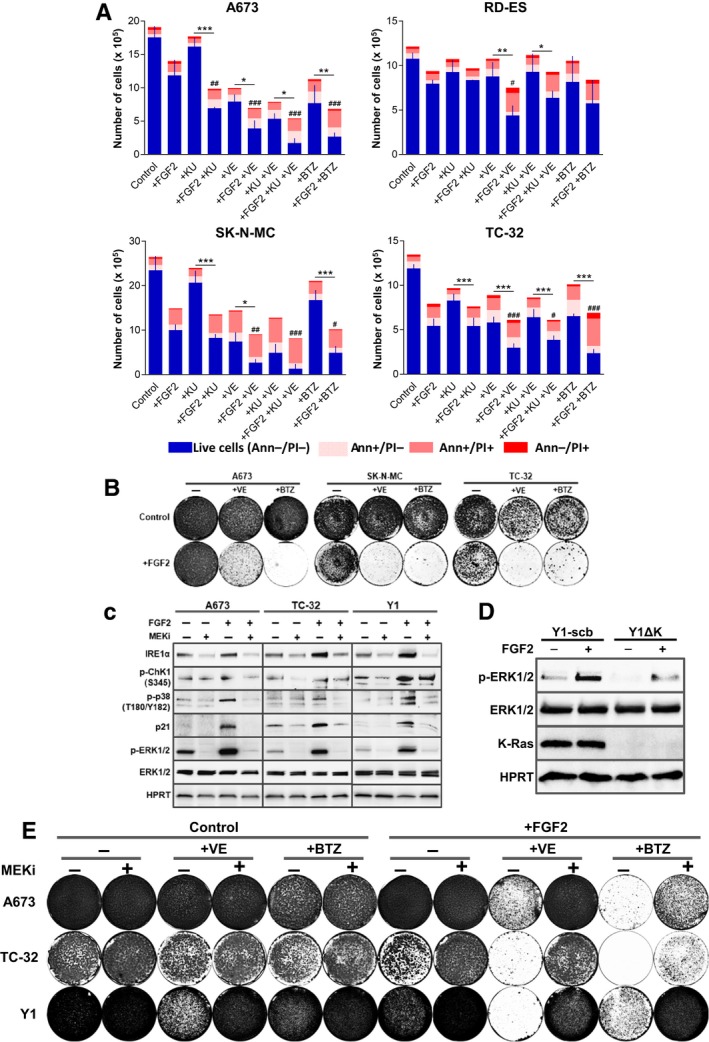
FGF2 promotes MAPK‐ERK1/2 sustained overactivation and lethally sensitizes human cancer cells to checkpoint and proteasome inhibition. (A) Flow cytometry data of ESFT cells growing in complete media in presence or absence of FGF2 for 72 h, with concomitant addition of KU‐55933 (+KU), VE‐821 (+VE), both (+KU +VE), or addition of bortezomib (+BTZ) in the last 48 h. Concentrations as follows: A673 cells FGF2 20 ng·mL^−1^, KU 5 μm, VE 5 μm, and BTZ 10 nm; 1 × 10^5^ cells were plated. RD‐ES cells FGF2 20 ng·mL^−1^, KU 5 μm, VE 5 μm, and BTZ 10 nm; 2.5 × 10^5^ cells were plated. SK‐N‐MC cells FGF2 1 ng·mL^−1^, KU 5 μm, VE 2 μm, and BTZ 10 nm; 2.5 × 10^5^ cells were plated. TC‐32 cells FGF2 5 ng·mL^−1^, KU 5 μm, VE 2 μm, and BTZ 10 nm; 1.5 × 10^5^ cells were plated. Annexin V/PI double stain was used to address cell death. Results are expressed in absolute numbers of cells per plate 72 h after stimulation. Error bars indicate mean ± SD of live cells (*n* = 3, from independent experiments). Asterisks indicate statistically significant differences. *P < 0.05, **P < 0.01 and ***P ≤ 0.001 (one‐way ANOVA of variance followed by multiple comparison post‐test). ^#^Significant differences from FGF2‐treated sample. ^#^ *P* < 0.05, ^##^ *P* < 0.01 and ^###^ *P* ≤ 0.001. (B) Representative assays comparing the long‐term viability of A673, TC‐32, and SK‐N‐MC cells treated with the combinations of FGF2 and VE‐821 or bortezomib. Cells were plated and treated as described in (A). After the treatments, the stimuli were washed out, the plates were grown in complete media for additional 10 days, and then fixed/stained. (C) Western blots comparing the levels of phospho‐ERK1/2 (p‐ERK1/2) and the stress markers IRE1α, phosphorylated Chk1 (p‐Chk1), phosphorylated p38 MAPK (p‐p38), and p21 in A673, TC‐32, and Y1 cells in presence or absence of FGF2 and the MEK inhibitor selumetinib. FGF2 (+) (20 ng·mL^−1^ for A673; 5 ng·mL^−1^ for TC‐32 and 10 ng·mL^−1^ for Y1 cells) was added to cells growing at complete media and 5 μm selumetinib was added to the indicated plates (MEKi +) 8 h after FGF2 addition. Plates were harvested 24 h after FGF2 addition. Total ERK and HPRT were used as loading controls. (D) Western blots comparing the levels of phospho‐ERK1/2 (p‐ERK1/2) and K‐Ras expression among Y1‐scb and Y1ΔK cells in the presence or absence of FGF2. FGF2 (+) (10 ng·mL^−1^) was added to cells growing at complete media and plates were harvested 24 h later. Total ERK and actin were used as loading controls. (E) Representative assays comparing the long‐term viability of A673, TC‐32, and Y1 cells treated with the combinations of FGF2 and VE‐821 or bortezomib, with or without the addition of the MEK inhibitor selumetinib. Cells were plated and treated as described in (A). Eight hour after FGF2 stimulation, 5 μm of selumetinib (MEKi +) was added to the indicated plates. Seventy‐two hour after FGF2 addition, the stimuli were washed out, the plates were grown in complete media for additional 10 days, and then fixed/stained.

The above data, focused on K‐Ras‐driven murine Y1 cancer cells, implied that mitogenic signaling activation combined with stress‐response pathways inhibitors could disrupt the homeostatic robustness of cancer cells resulting in cell death. Hence, we asked whether this hypothesis would hold true for human cancer cells. Cytostatic and cytotoxic effects of FGF2 over ESFT cells have been reported by different researchers in the last decades, with the specific molecular mechanisms of this toxicity varying among these studies (Passiatore *et al*., 2011; Schweigerer *et al*., 1987; Williamson *et al*., 2004). Thus, we tested A673, RD‐ES, SK‐N‐MC, and TC‐32 ESFT cells for the toxicities of these combinations of FGF2 and proteasome or checkpoint inhibitors. We focused on the potential of these regimens to kill cancer cells, irrespectively of the cell death subroutine engaged, as well as to reduce the number of viable cancer cells, using concentrations in which none of these stimuli induce pronounced cell death as a single agent. Thus, for each cell line, we plated the indicated number of cells, and after treatments, we measured cell death by Annexin V/propidium iodide double stain and the total number of cells in each sample by flow cytometry. For proteasome inhibition, we treated the cells with FGF2 for 24 h and then added bortezomib for additional 48 h before harvesting. The regimen for checkpoint inhibition was 72‐h treatment with FGF2 combined with ATM (KU‐55933), ATR (VE‐821) or both inhibitors, since the functions of these kinases can overlap but are not redundant (Cimprich and Cortez, 2008). Overall, the combination of FGF2 and proteasome or checkpoint inhibition increased the cell death and reduced the number of live cells to a greater extent than the respective stimuli as single agents in all four cancer cell models (Fig. 5A). For proteasome inhibition, striking results were found in A673, SK‐N‐MC and TC‐32 cells, in which the association with FGF2 reduced the number of live cells to about 1/3 of the observed for bortezomib alone (Fig. 5A). In all ESFT cells, the association of FGF2 and VE‐821 reduced the number of live cells to about half of the measured using this ATR inhibitor as a single agent (Fig. 5A). While the association of FGF2 and KU‐55933 resulted in significant increased toxicity only in A673 cells (Fig. 5A), in agreement with the role of ATR as the major player in the replication stress response (Saldivar *et al*., 2017).

We next addressed the effects of proteasome or ATR‐checkpoint inhibition on long‐term cell viability with or without FGF2. We treated A673, SK‐N‐MC, and TC‐32 cells as described above, washed out FGF2 and the inhibitors, and cultured the cells for additional 10 days. The results show minor or no effects of FGF2, bortezomib or VE‐821 after these times for all three cells. Conversely, the associations of FGF2 with these inhibitors were even more toxic than anticipated by the flow cytometry data, resulting in a massive reduction in cell viability after 10 days (Fig. 5B). These data demonstrated that FGF2 signaling activation can also sensitize human cancer cells to proteasome or checkpoint therapeutic inhibitors.

Ras, EWS‐FLI‐1, and many other driver oncogenes rely on aberrant MAPK‐ERK signaling pathway activation to promote tumorigenesis (Dhillon *et al*., 2007; Silvany *et al*., 2000). Thus, we argued whether further overactivation of this same pathway might underlie FGF2 toxicity and the observed increased mobilization and dependence on stress pathways. Our results showed that FGF2 signaling sustains higher levels of p‐ERK1/2 even 24 h after stimulation, comparing to control cells grown in complete media, in A673, TC‐32, and Y1 cells (Fig. 5C). This sustained MAPK‐ERK activation correlates with the upregulation of UPR (IRE1α) and DDR (p‐Chk1, p‐p38, and p21) markers (Fig. 5C). Pharmacological inhibition of MEK1/2 using the potent and selective inhibitor selumetinib, even 8 h after FGF2 stimulation, turned off sustained ERK activation and restored cell homeostasis (Fig. 5C). Coherently, K‐Ras depletion, which we showed above to protect from FGF2 toxicity and sensitization to proteasome and checkpoint inhibition, also prevented sustained MAPK overactivation in Y1ΔK cells (Fig. 5D). Finally, we used the same regimens described for Fig. 5B and added the MEK1/2 inhibitor 8 h after FGF2 stimulation. Disruption of FGF2‐induced sustained MAPK signaling alleviated or prevented the long‐term toxicity triggered by the combinations of FGF2 and ATR‐checkpoint or proteasome inhibition in A673, TC‐32, and Y1 cells (Fig. 5E).

Because we formerly reported that MAPK inhibition with the MEK inhibitor U0126 failed to prevent FGF2 toxicity in Y1 cells (Salotti *et al*., 2013), we next performed parallel western blots and viability assays using a third and highly potent MEK inhibitor, trametinib, to address these apparent discrepancies. The results showed that U0126 poorly alleviates the sustained MAPK‐ERK1/2 activation induced by FGF2 in Y1 and ESFT cells, comparing to selumetinib and trametinib (Fig. S1A). Moreover, corroborating the results using selumetinib (Fig. 5D), trametinib fully prevented the described toxicities in these cells, whereas U0126 provided partial or no protection (Fig. S1B, left). In addition, Williamson and coworkers proposed that p38 signaling underlies FGF2 toxicity in TC‐32 ESFT cells (Williamson *et al*., 2004). We now enquired whether p38 inhibition would prevent the cell death induced by combinations of FGF2 and VE‐821 or bortezomib in three ESFT cells (TC‐32, A673, and SK‐N‐MC), and also in Y1 cells. The results showed that p38 inhibition prevents the synergic toxicity of FGF2 and VE‐821, but not of FGF2 plus bortezomib in TC‐32 cells. Moreover, in the other two ESFT cell models and the murine Y1 cells, p38 inhibition failed to prevent toxicity; in fact, it synergized with bortezomib to enhance toxicity in SK‐N‐MC cells (Fig. S1B, right). These results point that MAPK‐ERK1/2, but not p38, signaling underlies the described toxicities in these malignant cells. Therefore, by sustaining MAPK‐ERK1/2 overactivation, a signaling pathway frequently overridden in malignant transformation, FGF2 reinforces the dependence on stress‐response pathways, increasing the toxicity of stress‐targeted therapeutic inhibitors in both murine and human cancer cells.

## 4 Discussion

Identification and effective targeting of stresses inherent to the malignant phenotype is a current goal in cancer research and therapy. The core rationale of this approach is that uncontrolled malignant proliferation comes with a cost: a stressed phenotype comprising a risky balance between antagonistic metabolic and molecular signaling pathways controlling homeostasis and viability. Therefore, both further induction and inhibition of stress‐response pathways can push cancer cells over an irreversible lethal threshold while sparing the normal cell counterparts. In this context, the results presented here highlight how mitogenic signaling can be manipulated to overload inherent stresses, disrupting the risky homeostatic robustness of cancer cells and sensitizing them to stress‐targeted therapies.

Many different mechanisms by which growth factors’ mitogenic signaling pathways contribute to the malignant progression have been emphasized in the cancer literature, and gain‐of‐function mutations along these mitogenic pathways are recognized driver oncogenic lesions in most human cancers. On the other hand, evidence is also accumulating showing that increasing mitogenic activation is not necessarily better for cancer cell fitness. For instance, EGFR and KRAS genes are frequently mutated in lung adenocarcinomas but with no overlap in individual samples. Unni *et al*. (2015) have recently shown that synthetic lethality rather than redundancy underlies this mutual exclusivity. In melanomas, some BRAF V600E‐driven tumors become resistant to the BRAF inhibitor vemurafenib through overexpression of BRAF V600E. In this context, withdrawal of the inhibitor resulted in tumor regression caused by a now over activated MAPK pathway (Thakur *et al*., 2013). These observations suggest that both inhibition and overactivation of canonically mitogenic pathways might disrupt tumor cell fitness.

As for other growth factors, pro‐tumor roles have been attributed to FGF2 signaling in different models and contexts (reviewed by Turner and Grose, 2010). However, most of these results are based on established cancer models, in which an optimal FGF2 signaling level was selected during malignant progression and is now part of its adapted and robust phenotype. Conversely, exogenous administration of FGF2 induced cytostatic or cytotoxic effects in breast cancer (Wang *et al*., 1998), Ewing's sarcoma family tumor (Williamson *et al*., 2004), and medulloblastoma (Fogarty *et al*., 2007) cell models among others. Moreover, transgenic mice overexpressing FGF2 in all major organs developed into old age showing no increased tumorigenesis (Coffin *et al*., 1995). Furthermore, regular subcutaneous injections of FGF2 also decreased or prevented xenograft tumor growth in mice without noticeable toxicity (Costa *et al*., 2008; Sturla *et al*., 2000). These observations suggest that, while FGF2 signaling can be pathologically overridden in certain cancers, exogenous FGF2 administration can disrupt cancer cell homeostasis both *in vivo* and *in vitro*.

Accordingly, our cell cycle kinetic analyses showed that FGF2 induces a general rather than phase‐specific arrest in Y1K‐Ras‐driven cancer cells. The observed delayed S‐phase entry and progression, G1 and G2 arrests incrementally contribute to FGF2 cytostatic effects in these cells. This cell cycle abnormal progression led to increased average cell size and protein concentration, implying that FGF2 signaling disrupts the homeostatic coupling between cell growth and proliferation in this model. It is known that EIF4E and S6K signaling play key roles in active protein synthesis and cell size control (Fingar *et al*., 2002). Thus, the resultant UPR activation is likely a consequence of the sustained EIF4E and S6K activity observed in FGF2‐treated cells. One of the strategies of UPR to mitigate proteotoxic stress is to downregulate protein synthesis, helping to restore protein homeostasis (Ron and Walter, 2007). By eliciting active protein synthesis during an ongoing UPR, FGF2 might push proteotoxic stress over the viability threshold. Proteotoxic stress is recognized as a potential Achilles’ heel of malignant cells. Strikingly, treatment of multiple myeloma with bortezomib may result in a complete response. This high sensitivity can be attributed to the extensive production of immunoglobulins by multiple myeloma cells, which accumulates due to bortezomib proteasome inhibition leading to a fatal proteotoxic stress (Meister *et al*., 2007; Obeng *et al*., 2006). This scenario provides a rationale for the observed induction of cell death triggered by the combination of FGF2 and bortezomib in these murine cancer cells. Noteworthily, FGF2 can also sensitize ESFT cells to bortezomib cytotoxicity. This panel of cancer cells was largely tolerant to 10 nm of bortezomib for 48 h. However, the results for A673, SK‐N‐MC, and TC‐32 cells, in which FGF2 or bortezomib alone had minor effects on long‐term cell viability but their association was highly toxic, highlight the therapeutic potential of this combination for inducing cancer cell death. It is promising because bortezomib can be very toxic to normal cells, limiting its therapeutic window (Chen *et al*., 2011). Additionally, K‐Ras‐depletion fully prevented the combined toxicity of FGF2 and bortezomib, linking the sensitizing effect of FGF2 to the malignant phenotype. Our results implicate that FGF2 signaling overactivation can efficiently disrupt proteostasis, resulting in a vulnerability common to cancer cells with diverse origins and driver oncogenic lesions.

The risky balance between oncogenic activity and increased mobilization of the DDR, frequently found in cancer cells, represents the other vulnerability which we explored here to target these malignant cell models. FGF2 induced stalling or collapse of DNA replication forks in Y1 cells along S‐phase. Similar replication stress has been shown to occur early in tumorigenesis, when the oncogenic activity causes increased firing of DNA replication origins leading to unscheduled S‐phase progression (Hills and Diffley, 2014). In this scenario, consequent DDR activation upregulating checkpoint proteins is an anticancer barrier that must be overcome in early malignant transformation (Bartkova *et al*., 2005; Gorgoulis *et al*., 2005). Y1 cells, like other malignant cells, displayed tonic levels of DDR activation which are compatible with high proliferation rates. It is noteworthy that FGF2 stimulation increased the activation of checkpoint proteins, reactivating this anticancer barrier and restraining cell proliferation in this K‐Ras‐driven model. By enhancing replication stress on these cells, FGF2 also increased their dependence on checkpoint activity for survival; hence, the combination of FGF2 stimulation and checkpoint abrogation triggered cell death. Importantly, we showed that the same approach is also effective to trigger cell death in the panel of ESFT cells. The combination of FGF2 and VE‐821, but not these agents alone, strongly reduced long‐term viability of A673, SK‐N‐MC, and TC‐32 cells. The increased dependency on DDR has been described in other cancer cells as an example of nononcogene addiction (Luo *et al*., 2009). In this regard, checkpoint inhibition has recently been shown to sensitize cancer cells to radiotherapy and chemotherapy based on DNA damaging agents (Huntoon *et al*., 2013; Prevo *et al*., 2012). However, both radiation and genotoxic agents are frequently very harmful also to nonmalignant cells. We provided here evidence that mitogenic signaling activation and checkpoint inhibition might represent an efficient combination stress overloading/sensitization to exploit nononcogene addiction in cancer therapies.

The specific molecular mechanisms of FGF2 toxicity and sensitization to stress‐targeted inhibitors likely vary among Y1 and ESFT cells and engage different cell death subroutines as observed in our data. However, sustained overactivation of the Ras‐MAPKs‐ERK1/2 axis by FGF2 is a common feature of the vulnerabilities which we emphasized here. K‐Ras depletion in Y1ΔK cells prevented MAPK‐ERK1/2 overactivation induced by FGF2. These cells showed no decrease in viability or proliferation rates in solid substrate, and no FGF2 toxicity or sensitization to proteasome or checkpoint inhibition. However, K‐Ras depletion suppressed malignant traits of these cells. These data indicate that the tuning of K‐Ras‐MAPKs activation, which underlies the proliferation and malignancy in these cells, likely is also the molecular target of FGF2 toxicity. In ESFT cells, EWS/FLI‐1 fusion protein suppresses Sprouty 1 expression, a negative‐feedback regulator of Ras‐MAPKs signaling downstream of FGF receptors; this genetic lesion was proposed to render unrestrained FGF2‐induced proliferation in these cells *in vitro* and *in vivo* (Cidre‐Aranaz *et al*., 2017). Indeed, constitutive activation of MAPK‐ERK1/2 was found in several ESFT cells, and a Ras dominant negative or MAPK‐ERK1/2 pharmacological inhibition restrained the transforming activity of EWS/FLI‐1 in immortalized fibroblasts (Silvany *et al*., 2000). Interestingly, FGF2 itself induces EWS/FLI‐1 expression in ESFT cells (Girnita *et al*., 2000). Taken together, these data suggest that, at optimal growth conditions, exogenous FGF2 likely induces a positive feedback loop resulting in sustained and toxic MAPK‐ERK1/2 overactivation in these cells. This scenario is supported by our data showing not only that FGF2 induced sustained higher levels of active ERK1/2, but also that MAPK inhibition, even 8 h after FGF2 stimulus, restored cell homeostasis and rescued ESFT and Y1 cells from the synergic toxicities which we described above.

The data and the background discussed here argue the question of whether, contraintuitively, growth factors signaling activation might be clinically explored in cancer therapies. While this major question cannot be exhausted in the scope of this current work, the data provided here show that FGF2 can efficiently disturb the homeostasis of cancer cells from different origin and phenotypes, increasing the toxicity of checkpoint and proteasome inhibitors. Importantly, because we focused here on the sensitizing effect of FGF2, we used doses and times in which neither FGF2 nor the inhibitors trigger massive cell death as a single agent. This implies that the overall toxicity of these combinations over cancer cells can be further improved by tailoring the regimens.

## 5 Conclusions

Our data provide evidence that additional stimulation of the same signaling pathways overridden by the malignant transformation might further increase the mobilization and dependence on stress‐response pathways in cancer cells, hence improving the efficacy and selectivity of stress‐targeted therapies. This approach might be particularly useful at relapsed tumors resulting from acquired resistance to MAPK‐ERK1/2 inhibitors, but also provides a potential game‐changing novel therapeutic perspective for other human cancers.

## Conflict of interest

The authors declare no conflict of interest.

## Author contributions

MHD, CSF, and HAA conceived the rationale of the experimental design and this manuscript, with fundamental insights from MSR and VN. MHD, CSF, LLA, MSS, ECL, and EOS carried out the experiments. JDZ performed the statistical analyses. MHD wrote the manuscript with essential contribution from CSF and JDZ; IAP and HAA guided the writing of the manuscript and edited the manuscript; all authors read and approved the final version of the manuscript. IAP and HAA supervised the project.

## Supporting information


**Fig. S1.** The tuning of MAPK‐ERK1/2, but not p38 signaling underlies FGF2 sensitization to ATR‐checkpoint or proteasome inhibition in murine K‐Ras‐driven and ESFT cancer cells.

## References

[mol212402-bib-0001] Armelin HA (1973) Pituitary extracts and steroid hormones in the control of 3T3 cell growth. Proc Natl Acad Sci USA 70, 2702–2706. 4354860 10.1073/pnas.70.9.2702 PMC427087

[mol212402-bib-0002] Bartkova J , Horejsí Z , Koed K and Dramer A (2005) DNA damage response as a candidate anti‐cancer barrier in early human tumorigenesis. Nature 434, 864. 15829956 10.1038/nature03482

[mol212402-bib-0003] Biswas B and Bakhshi S (2016) Management of Ewing sarcoma family of tumors: current scenario and unmet need. World J Orthop 7, 527. 27672565 10.5312/wjo.v7.i9.527 PMC5027007

[mol212402-bib-0004] Cavaletti G and Jakubowiak AJ (2010) Peripheral neuropathy during bortezomib treatment of multiple myeloma: a review of recent studies. Leuk Lymphoma 51, 1178–1187. 20497001 10.3109/10428194.2010.483303

[mol212402-bib-0005] Chen D , Frezza M , Schmitt S , Kanwar J and Dou PQ (2011) Bortezomib as the first proteasome inhibitor anticancer drug: current status and future perspectives. Curr Cancer Drug Targets 11, 239–253. 21247388 10.2174/156800911794519752 PMC3306611

[mol212402-bib-0006] Cidre‐Aranaz F , Grünewald TG , Surdez D , García‐García L , Carlos Lázaro J , Kirchner T , González‐González L , Sastre A , García‐Miguel P , López‐Pérez SE *et al* (2017) EWS‐FLI1‐mediated suppression of the RAS‐antagonist Sprouty 1 (SPRY1) confers aggressiveness to Ewing sarcoma. Oncogene 36, 766–776. 27375017 10.1038/onc.2016.244

[mol212402-bib-0007] Cimprich KA and Cortez D (2008) ATR: an essential regulator of genome integrity. Nat Rev Mol Cell Biol 9, 616. 18594563 10.1038/nrm2450 PMC2663384

[mol212402-bib-0008] Coffin JD , Florkiewicz RZ , Neumann J , Mort‐Hopkins T , Dorn GW 2nd , Lightfoot P , German R , Howles PN , Kier A and O'Toole BA (1995) Abnormal bone growth and selective translational regulation in basic fibroblast growth factor (FGF‐2) transgenic mice. Mol Biol Cell 6, 1861–1873. 8590811 10.1091/mbc.6.12.1861 PMC301338

[mol212402-bib-0009] Costa ET , Forti FL , Matos TG , Dermargos A , Nakano F , Salotti J , Rocha KM , Asprino PF , Yoshihara CK , Koga MM *et al* (2008) Fibroblast growth factor 2 restrains Ras‐driven proliferation of malignant cells by triggering RhoA‐mediated senescence. Cancer Res 68, 6215–6223. 18676845 10.1158/0008-5472.CAN-08-0342

[mol212402-bib-0010] Csizmar CM , Kim DH and Sachs Z (2016) The role of the proteasome in AML. Blood Cancer J 6, e503. 27911437 10.1038/bcj.2016.112 PMC5223148

[mol212402-bib-0011] Dhillon AS , Hagan S , Rath O and Kolch W (2007) MAP kinase signalling pathways in cancer. Oncogene 26, 3279–3290. 17496922 10.1038/sj.onc.1210421

[mol212402-bib-0012] Fingar DC , Salama S , Tsou C , Harlow ED and Blenis J (2002) Mammalian cell size is controlled by mTOR and its downstream targets S6K1 and 4EBP1/eIF4E. Genes Dev 16, 1472–1487. 12080086 10.1101/gad.995802 PMC186342

[mol212402-bib-0013] Fogarty MP , Emmenegger BA , Grasfeder LL , Oliver TG and Wechsler‐Reya RJ (2007) Fibroblast growth factor blocks Sonic hedgehog signaling in neuronal precursors and tumor cells. Proc Natl Acad Sci USA 104, 2973–2978. 17299056 10.1073/pnas.0605770104 PMC1815291

[mol212402-bib-0014] Gagou ME , Zuazua‐Villar P and Meuth M (2010) Enhanced H2AX phosphorylation, DNA replication fork arrest, and cell death in the absence of Chk1. Mol Biol Cell 21, 739–752. 20053681 10.1091/mbc.E09-07-0618 PMC2828961

[mol212402-bib-0015] Gaspar N , Hawkins DS , Dirksen U , Lewis IJ , Ferrari S , Le Deley M‐C , Kovar H , Grimer R , Whelan J , Claude L *et al* (2015) Ewing sarcoma: current management and future approaches through collaboration. J Clin Oncol 33, 3036–3046. 26304893 10.1200/JCO.2014.59.5256

[mol212402-bib-0016] Girnita L , Girnita A , Wang M , Meis‐Kindblom JM , Kindblom LG and Larsson O (2000) A link between basic fibroblast growth factor (bFGF) and EWS/FLI‐1 in Ewing's sarcoma cells. Oncogene 19, 4298. 10980604 10.1038/sj.onc.1203755

[mol212402-bib-3000] Gorgoulis VG , Vassiliou LV , Karakaidos P , Zacharatos P , Kotsinas A , Liloglou T , Venere M , DiTullio Jr RA , Kastrinakis NG , Levy B *et al* (2005) Activation of the DNA damage checkpoint and genomic instability in human precancerous lesions. Nature 434, 907. 15829965 10.1038/nature03485

[mol212402-bib-0017] Harper JW , Elledge SJ (2007) The DNA damage response: ten years after. Mol Cell, 28, 739–745. 18082599 10.1016/j.molcel.2007.11.015

[mol212402-bib-0018] Hickson I , Zhao Y , Richardson CJ , Green SJ , Martin NM , Orr AI , Reaper PM , Jackson SP , Curtin NJ and Smith GC (2004) Identification and characterization of a novel and specific inhibitor of the ataxia‐telangiectasia mutated kinase ATM. Cancer Res 64, 9152–9159. 15604286 10.1158/0008-5472.CAN-04-2727

[mol212402-bib-0019] Hills SA and Diffley JF (2014) DNA replication and oncogene‐induced replication stress. Curr Biol 24, R435–R444. 24845676 10.1016/j.cub.2014.04.012

[mol212402-bib-0020] Huntoon CJ , Flatten KS , Hendrickson AEW , Huehls AM , Sutor SL , Kaufmann SH and Karnitz LM (2013) ATR inhibition broadly sensitizes ovarian cancer cells to chemotherapy independent of BRCA status. Cancer Res 73, 3683–3691. 23548269 10.1158/0008-5472.CAN-13-0110 PMC3687010

[mol212402-bib-0021] Itoh N , Ornitz DM (2011) Fibroblast growth factors: from molecular evolution to roles in development, metabolism and disease. J Biochem, 149, 121–130. 20940169 10.1093/jb/mvq121 PMC3106964

[mol212402-bib-0022] Kalal BS , Upadhya D , Pai VR (2017) Chemotherapy resistance mechanisms in advanced skin cancer. Oncol Rev, 11, 326. 28382191 10.4081/oncol.2017.326 PMC5379221

[mol212402-bib-0023] Luo J , Solimini NL and Elledge SJ (2009) Principles of cancer therapy: oncogene and non‐oncogene addiction. Cell 136, 823–837. 19269363 10.1016/j.cell.2009.02.024 PMC2894612

[mol212402-bib-0024] Meister S , Schubert U , Neubert K , Herrmann K , Burger R , Gramatzki M , Hahn S , Schreiber S , Wilhelm S , Herrmann M *et al* (2007) Extensive immunoglobulin production sensitizes myeloma cells for proteasome inhibition. Cancer Res 67, 1783–1792. 17308121 10.1158/0008-5472.CAN-06-2258

[mol212402-bib-0025] Neznanov N , Komarov AP , Neznanova L , Stanhope‐Baker P and Gudkov AV (2011) Proteotoxic stress targeted therapy (PSTT): induction of protein misfolding enhances the antitumor effect of the proteasome inhibitor bortezomib. Oncotarget 2, 209. 21444945 10.18632/oncotarget.246 PMC3260823

[mol212402-bib-0026] Obeng EA , Carlson LM , Gutman DM , Harrington WJ , Lee KP and Boise LH (2006) Proteasome inhibitors induce a terminal unfolded protein response in multiple myeloma cells. Blood 107, 4907–4916. 16507771 10.1182/blood-2005-08-3531 PMC1895817

[mol212402-bib-0027] Passiatore G , Gentilella A , Rom S , Pacifici M , Bergonzini V and Peruzzi F (2011) Induction of Id‐1 by FGF‐2 involves activity of EGR‐1 and sensitizes neuroblastoma cells to cell death. J Cell Physiol 226, 1763–1770. 21506108 10.1002/jcp.22505 PMC3760689

[mol212402-bib-0028] Prevo R , Fokas E , Reaper PM , Charlton PA , Pollard JR , McKenna WG , Muschel RJ and Brunner TB (2012) The novel ATR inhibitor VE‐821 increases sensitivity of pancreatic cancer cells to radiation and chemotherapy. Cancer Biol Ther 13, 1072–1081. 22825331 10.4161/cbt.21093 PMC3461814

[mol212402-bib-0029] Prior IA , Lewis PD and Mattos C (2012) A comprehensive survey of Ras mutations in cancer. Cancer Res 72, 2457–2467. 22589270 10.1158/0008-5472.CAN-11-2612 PMC3354961

[mol212402-bib-0030] Reaper PM , Griffiths MR , Long JM , Charrier JD , MacCormick S , Charlton PA , Golec JMC and Pollard JR (2011) Selective killing of ATM‐or p53‐deficient cancer cells through inhibition of ATR. Nat Chem Biol 7, 428–430. 21490603 10.1038/nchembio.573

[mol212402-bib-0031] Ron D and Walter P (2007) Signal integration in the endoplasmic reticulum unfolded protein response. Nat Rev Mol Cell Biol 8, 519. 17565364 10.1038/nrm2199

[mol212402-bib-0032] Saldivar JC , Cortez D , Cimprich KA (2017) The essential kinase ATR: ensuring faithful duplication of a challenging genome. Nat Rev Mol Cell Biol 18, 622–636. 28811666 10.1038/nrm.2017.67 PMC5796526

[mol212402-bib-0033] Salotti J , Dias MH , Koga MM and Armelin HA (2013) Fibroblast growth factor 2 causes G2/M cell cycle arrest in ras‐driven tumor cells through a Src‐dependent pathway. PLoS One 8, e72582. 23991123 10.1371/journal.pone.0072582 PMC3753234

[mol212402-bib-0034] Sanjana NE , Shalem O and Zhang F (2014) Improved vectors and genome‐wide libraries for CRISPR screening. Nat Methods 11, 783. 25075903 10.1038/nmeth.3047 PMC4486245

[mol212402-bib-0035] Schwab M , Alitalo K , Varmus HE , Bishop JM and George D (1983) A cellular oncogene (c‐Ki‐ras) is amplified, overexpressed and located within karyotypic abnormalities in mouse adrenocortical tumour cells. Nature 303, 497–501. 6304530 10.1038/303497a0

[mol212402-bib-0036] Schweigerer L , Neufeld G and Gospodarowicz D (1987) Basic fibroblast growth factor as a growth inhibitor for cultured human tumor cells. J Clin Invest 80, 1516. 2824563 10.1172/JCI113236 PMC442414

[mol212402-bib-0037] Schwindt TT , Forti FL , Juliano MA , Juliano L and Armelin HA (2003) Arginine vasopressin inhibition of cyclin D1 gene expression blocks the cell cycle and cell proliferation in the mouse Y1 adrenocortical tumor cell line. Biochemistry 42, 2116–2121. 12590600 10.1021/bi026807g

[mol212402-bib-0038] Silvany RE , Eliazer S , Wolff NC and Ilaria RL (2000) Interference with the constitutive activation of ERK1 and ERK2 impairs EWS/FLI‐1‐dependent transformation. Oncogene 19, 4523. 11002425 10.1038/sj.onc.1203811

[mol212402-bib-0039] Singh A , Greninger P , Rhodes D , Koopman L , Violette S , Bardeesy N and Settleman J (2009) A gene expression signature associated with “K‐Ras addiction” reveals regulators of EMT and tumor cell survival. Cancer Cell 15, 489–500. 19477428 10.1016/j.ccr.2009.03.022 PMC2743093

[mol212402-bib-0040] Sonenberg N and Hinnebusch AG (2009) Regulation of translation initiation in eukaryotes: mechanisms and biological targets. Cell 136, 731–745. 19239892 10.1016/j.cell.2009.01.042 PMC3610329

[mol212402-bib-0041] Sturla LM , Westwood G , Selby PJ , Lewis IJ and Burchill SA (2000) Induction of cell death by basic fibroblast growth factor in Ewing's sarcoma. Cancer Res 60, 6160–6170. 11085540

[mol212402-bib-0042] Swift LH and Golsteyn RM (2014) Genotoxic anti‐cancer agents and their relationship to DNA damage, mitosis, and checkpoint adaptation in proliferating cancer cells. Int J Mol Sci 15, 3403–3431. 24573252 10.3390/ijms15033403 PMC3975345

[mol212402-bib-0043] Thakur MD , Salangsang F , Landman AS , Sellers WR , Pryer NK , Levesque MP , Dummer R , McMahon M and Stuart DD (2013) Modelling vemurafenib resistance in melanoma reveals a strategy to forestall drug resistance. Nature 494, 251. 23302800 10.1038/nature11814 PMC3930354

[mol212402-bib-0044] Toomey EC , Schiffman JD and Lessnick SL (2010) Recent advances in the molecular pathogenesis of Ewing's sarcoma. Oncogene 29, 4504. 20543858 10.1038/onc.2010.205 PMC3555143

[mol212402-bib-0045] Turner N , Grose R (2010) Fibroblast growth factor signalling: from development to cancer. Nat Rev Cancer, 10, 116. 20094046 10.1038/nrc2780

[mol212402-bib-0046] Unni AM , Lockwood WW , Zejnullahu K , Lee‐Lin SQ and Varmus H (2015) Evidence that synthetic lethality underlies the mutual exclusivity of oncogenic KRAS and EGFR mutations in lung adenocarcinoma. Elife 4, e06907. 26047463 10.7554/eLife.06907 PMC4478584

[mol212402-bib-0047] Walter P and Ron D (2011) The unfolded protein response: from stress pathway to homeostatic regulation. Science 334, 1081–1086. 22116877 10.1126/science.1209038

[mol212402-bib-0048] Wang Q , Maloof P , Wang H , Fenig E , Stein D , Nichols G , Denny TN , Yahalom J and Wieder R (1998) Basic fibroblast growth factor downregulates Bcl‐2 and promotes apoptosis in MCF‐7 human breast cancer cells. Exp Cell Res 238, 177–187. 9457070 10.1006/excr.1997.3820

[mol212402-bib-0049] Williamson AJ , Dibling BC , Boyne JR , Selby P and Burchill SA (2004) Basic fibroblast growth factor‐induced cell death is effected through sustained activation of p38MAPK and up‐regulation of the death receptor p75NTR. J Biol Chem 279, 47912–47928. 15310753 10.1074/jbc.M409035200

