# Supplementary figures and images for: Fibroblast Growth Factor 2 lethally sensitizes cancer cells to stress‐targeted therapeutic inhibitors
Source: Mol Oncol. 2018 Dec 3;13(2):290–306. doi: 10.1002/1878-0261.12402 (PMC6360366; doi:10.1002/1878-0261.12402)

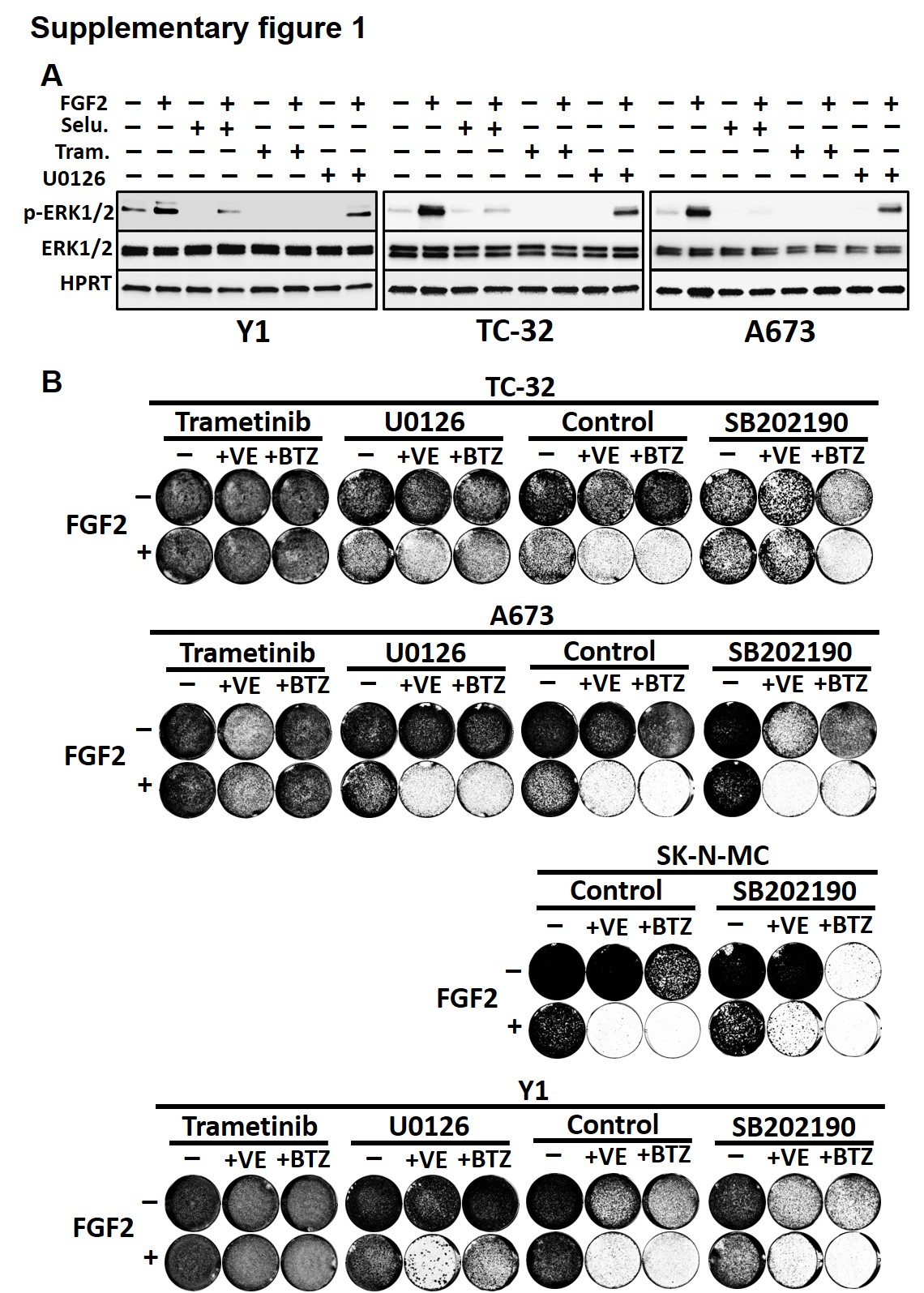

Supplement: Supplementary file 1 — Fig. S1. The tuning of MAPK‐ERK1/2, but not p38 signaling underlies FGF2 sensitization to ATR‐checkpoint or proteasome inhibition in murine K‐Ras‐driven and ESFT cancer cells. [file MOL2-13-290-s001.jpg]
